# Ten simple rules for scientists getting started in science education

**DOI:** 10.1371/journal.pcbi.1009556

**Published:** 2021-12-02

**Authors:** Stephen Pompea, Pedro Russo

**Affiliations:** 1 NSF’s NOIRLab, Tucson, Arizona, United States of America; 2 James C. Wyant College of Optical Sciences, University of Arizona, Tucson, Arizona, United States of America; 3 Department of Astronomy and Steward Observatory, University of Arizona, Tucson, Arizona, United States of America; 4 Leiden Observatory, Leiden University, Leiden, the Netherlands; 5 Department of Science Communication & Society and Leiden Observatory, Leiden University, Leiden, the Netherlands

## Introduction

Scientists from research institutes, universities, and government labs can participate in diverse education efforts with schools, museums, after-school programs, clubs, and affinity groups such as scouts. Researchers often feel that they are well prepared to engage with the educational system by virtue of having been students, parents, lecturers, university course creators, and museum goers. However, it needs to be remembered that being on the receiving end of a service (e.g., as a student or a medical patient) is not always sufficient preparation for being on the delivering end (as a classroom teacher or a medical doctor), especially when new challenges and approaches are needed. The Dunning–Kruger effect may also be operating. This is where a person’s knowledge in areas outside their expertise is more limited than they appreciate, as is their own understanding of what they do not know.

We are 2 scientists working in dozens of countries specializing in the design, delivery, and evaluation of education and community engagement (ECE) programs in scientific research organizations [1]. Over decades, we have appreciated working in the field of ECE with many scientists who have excellent communication skills, broad perspectives, and oodles of enthusiasm, energy, and idealism. Most scientists, however, still need additional guidance and training in their roles as education program participants or as supporters, managers, and promoters of these programs. Fortunately, these programs are becoming more common. Programs such as Portal to the Public, with empirical evidence for their effectiveness, are designed to remediate this situation by providing professional development for scientists participating in museum programs [2].

We rely on research that evaluates evidence-based practices to inform our work in the challenging area of improving science education practices in complex system with deep needs and some long-standing problems.

The National Aeronautics and Space Administration (NASA) astronaut, George “Pinky” Nelson, who has worked both in science research and in science education reform, described the latter effort as “It is not rocket science—it is much harder!” [3]. From our experiences both in research and development in university and industry settings and in working to improve the science education system worldwide, we heartily agree with his summary!

These 10 rules can make it easier for you as a scientist to work productively and efficiently in educational activities, minimizing your frustration and maximizing impact and personal satisfaction. The following 10 rules blend our knowledge of the research literature with the most important lessons learned from our decades-long practices of creating and running well-evaluated projects that field-test evidence-based science education practices and strategies.

## Rule 1: Examine your motivations

An examination of your motivation for getting involved is an important first step. Doing this will provide clarity on your intentions and help create realistic expectations. Not all of these questions will be answerable immediately:

- What are my motives?
- What do I want to achieve and my audience to take with them?
- What history and experience do I bring to this effort? How did I get interested in this?
- Who do I want to serve or connect with more strongly? What areas do I feel passionately about?
- How much time do I want to invest now and in the long term?
- Am I willing to learn new skills and grow in this role?

## Rule 2: Understand your organization’s perspective

Your efforts in science education can be done after work and on a completely voluntary basis, unconnected to your work roles. However, many scientists want to leverage support from their organization as they assist in educational settings. If this is your case, an analysis of your organization’s perspective and potential level of support is useful. These questions may be helpful:

- Is there organizational support in the form of encouragement, training, educational supplies, or transportation assistance?
- Is this effort part of your job or work assignment, or is it an add-on to your normal duties?
- Is there an office in your organization that coordinates and encourages educational efforts? Are there people who can help you, arrange roles, or give you advice?
- Will your efforts in this area receive acknowledgments or rewards such as promotions and pay raises? Are they of value to the larger organization or to external funders?
- What are the organizational barriers that make your entry to this area or the support of your efforts more challenging?

## Rule 3: Assist the educational ecosystem

It is natural that after many years in the science field you love, you will want to help others to have similar educational experiences. You may even feel strongly that your personal experience or insights can be valuable in reforming the educational system. You are ready to intervene! Before you do that, imagine how you would feel if somebody showed up at your place of work and said that they have been thinking for a long time about how to improve what you do. Would you mind if they took charge of your work, so that they can do it their “better way”? How would you react to their claim that they can make your work happen much more productively?

It is important to appreciate that educators in informal science education settings such as museums and in formal education settings such as schools are part of a larger educational system with many conflicting demands and expectations placed on them. They are often lacking the resources and support they need to facilitate change. In museums, attendance levels and funding issues play a dominant role. In schools, curriculum and the high-stakes testing of students can create extreme pressure on teachers to teach in ways they do not professionally embrace. The tentative job security of most educators can also make them feel threatened by outsiders who may not approve of what they are doing and may be vocal in their disapproval (e.g., to the museum director or the principal). Many science educators do not have the strong science backgrounds they would like to have. Many are working out of their main field of study. Nearly all museums and schools have a shortage of equipment for science experiments, demonstrations, and other active learning activities.

The process of science is often not well known to science educators. Unlike you, they are not science practitioners participating in the science enterprise day after day for years. Educators (even specialized science educators) have a very different status, level of autonomy, training, and working conditions than scientists. Museums and schools each have a quite different working culture than research institutions (and from each other).

Given these considerations, a starting point for your work ought to be to help them succeed better as science educators, rather than for you to critique their approach, content knowledge, educational materials and exhibits, or pedagogy. First, find out from the educator you are working with what they need or want from you and then work to provide it. Don’t try to “fix” the museum or school. You will be surprised at how much you can help with your simple efforts and encouragement.

## Rule 4: Be a guide on the side

Scientists often make museum and school visits, are guest speakers talking about their own work, and serve as role models standing in front of their audiences. Sometimes, they also bring a demonstration or activity. These efforts can have value, especially if discussed in detail with the educator ahead of time, so the educator can integrate the presentation with what is being studied. However, there are many other ways scientists can contribute, including facilitation and advocacy roles. They can help arrange or lead field trips, assist science research projects (including citizen science projects), connect museum and school groups to bigger problem-solving efforts, create access to data, acquire teaching resources, and serve as vocal advocates for science educators, to name a few.

Progressive educators and scientists work to not be at the front in charge, “the sage on a stage,” but rather to be “the guide on the side.” You especially can contribute to a better understanding of “inquiry-based” learning processes that illustrate the way scientists do science. To play an effective role in any support role, you will need to form mutually respectful, collegial relationships with educators that acknowledge that their expertise in education is equal and complementary to the value of the scientist’s expertise in science.

Pretertiary education is quite different in its approach than university education. Most people who have taught at many levels (including us) believe that the younger the students, the more challenging it becomes to teach. For younger students, new skills are also especially needed since teaching is not about “telling” people what you know. An understanding of different learning styles, child and adolescent psychology, the intricacies of concept development at different ages, and how to work with misconceptions and naive theories can also play significant roles in working with these younger students. Working with a group of young children is not easy and requires much more than enthusiasm and a high energy level. It requires specialized training to be effective.

Having a close friend or partner who is a long-time educator can certainly sensitize a scientist to the joys and hardships of working in the education area. However, even this relationship does not provide the requisite background for teaching at this level any more than having a partner who is a gastroenterologist enables you to do a colonoscopy (legal considerations aside, in both cases). Scientists often underestimate the difficulties of teaching diverse groups in the pretertiary education environment and are often overconfident of their education skills. You will be most effective when you collaborate rather than compete with educators and develop a respect for the less obvious skills of educators and of their understated self-representation of their own expertise and experience.

## Rule 5: Partner up!

If your organization is lucky enough to have its own ECE team or a department devoted to this, your entry and participation in the educational arena can be made much easier. These professionals probably have formed partnerships with educational providers and have developed a deeper understanding of your community’s needs; both of these can facilitate your entry into this arena. Note that ECE professionals are different from those who serve the communications or public relations initiatives of your organization. ECE professionals can evaluate your skills, interests, motivations, and personality to help you find appropriate roles of value to the educational system.

If you don’t have an ECE department in your organization, then you may have to find and develop educational partnerships yourself. This often isn’t easy and can have a high activation energy. Education professionals are often polite to well-meaning, but ignorant outsiders who want to help them. That civility should not be taken as a validation of the efficacy of your efforts.

In summary, your ideas need a willing partner in order for them to be implemented successfully. It is your job to find those educational partners, to convince them of your good will and sincerity, and to learn how you can help them. It will not be generally productive for you to proceed unilaterally without cultivating, listening to, and working with partners.

## Rule 6: Go beyond the deficit model

Scientists often adopt a deficit model to guide their understanding of how to improve science education. They believe that the audience is missing something that they can provide or that the educational system has broken parts that are fixable by scientists.

Your formation of a deficit model might have begun with the realization that college students haven’t mastered a key science concept. You, the scientist, want to help fix this problem. The blame may be on the course the students took earlier or even the conditions much earlier in their schooling. Their former teachers might also be viewed as part of the problem. It is natural for the scientist to feel that something is lacking and to want to fix that problem in a direct way by filling in the perceived gap. The real issues behind the problem may be much more complicated and can’t always be fixed by filling the teacher or student with more knowledge.

Scientists often believe that the more you know, the better you are as an educator. This is an expression of a knowledge transmission model that can be crudely summarized as “you talk, they listen and learn.” Many college instructors have discovered that is not true that the better you are at presenting, the more they learn. Students learn best when more active learning approaches are used [4].

Holding these deficit-based models and their underlying assumptions do not aid you in working with younger learners who are novices, rather than the expert learners you are used to, such as college students majoring in science. This can lead to rookie mistakes: the university researcher going to a secondary school classroom to present 50 PowerPoint slides on their research or that same scientist assuming that they were following the teacher’s request for a hands-on activity by handing around an object from their work so that students could touch it. Novice learners do not have the extensive knowledge and the concept scaffold to learn in these ways. Scientists can improve their pedagogical approaches, just as teachers can learn more about scientific principles and approaches. Both can learn, especially with each other’s help.

## Rule 7: Learn and use best practices

Certain skills, approaches, and best practices are needed to work effectively in educational settings. Your enthusiasm, intelligence, and science experience can carry you only so far in being an effective educator. How can you develop a broader understanding of which practices work or don’t work? First, please convince yourself that a polite student in the front row nodding their head as you speak in a classroom is not an acknowledgment of your teaching expertise. Perhaps, if you were not there, the students would be relegated to answering the questions at the back of the chapter. Their nods simply concede that you are a bit more exciting than that! If you can apply some best practices, the student and other learners will be even more grateful and will learn more!

How can you learn about the research on best educational practices? First, realize that the educational literature is quite well developed in this area with many general and discipline specific resources. The United States National Academies of Sciences, Engineering, and Medicine has free, fully downloadable, authoritative, and well-written books on learning science in informal environments [5], school learning, citizen science, teacher professional development, university learning, and learning in specific science disciplines [6].

## Rule 8: Cultivate new roles

There are many interesting roles and forms of advocacy available to you. Many of them are more fun than traditional roles. For example, “giving a talk about my research work” can take many forms. A formal public lecture at an esteemed institution or museum may look great on your CV, but may lack any real time for discussion or making a connection with the audience. A much shorter and less formal talk at a pub may be more rewarding. It will also allow you to explore interactions with the science-interested public with a much smaller power differential between you and your audience. There will much more time for personal questions, reflections, and mutual discussion and sharing. This may be more satisfying and a more powerful learning experience for you. There are many nuances to the interaction of scientists with the public; be aware of them [7].

Although it is exciting to be a celebrity and the center of attention, try relaxing and blending into a supporting role at events and programs. Scientists are often portrayed in a negative light by the media (e.g., egotistical, overbearing, and one-dimensional). See if you can help develop a new image of scientists by your behavior in some useful, but different roles.

## Rule 9: Appreciate the value of “informal” education

It is easy to think of education as synonymous with schools or as professional development and training efforts with teachers that expand their knowledge. There are many other educational venues apart from schools that serve families and lifelong learners and which appreciate the efforts of scientists [8]. These include museums, libraries, science and technology centers, botanical gardens, nature centers, zoos, aquaria, and parks. Educational settings of great value also include after school programs, clubs, and groups that meet at neighborhood and community centers. There are many ways to assist and partner with these “informal” or “free choice” educational institutions and to assist their programs. For example, libraries often have after school and weekend youth activities for students of all ages. These programs are responsive to the needs of the many students who are looking to fill the significant gap between the end of the school day and when their parents return home from work. Many of these free choice venues do not emphasize science education, due to the lack or training or equipment to conduct these programs. You can help them enormously.

The same process of getting to know and understand the needs of classroom educators applies to these informal education professionals. You can work to become a trusted and reliable partner to them. Asking them “Do you want me to implement my pet project, or shall I present my work at your facility?” is probably not asking the right question. (Sorry for repeating this once again!) Resource-hungry youth leaders and their institutions almost invariably answer a polite “yes” to this sort of question (unfortunately!). The better questions for you to ask are the following: “What do you do right now?,” “What do you need now to help you do that better?,” and “How can I help you get what you need?.” Later in the relationship might be the appropriate time to explore the value of projects related to your specific interests.

## Rule 10: Be open to making mistakes

The theme that underlies these rules is that helping in education is a new area for you as a scientist that requires some new skills and approaches in order for you to be most effective. While some scientists feel that they have already have everything they need for efforts in this area, many others might feel out of their comfort zone as they understand better the complex needs and realities of museums, schools, and other education settings. After your initial efforts, you may feel that you are ill-suited for additional education roles even though your motivation to help is still strong. In order to feel comfortable in these new settings, cultivating a more open, tolerant, and even forgiving mindset is valuable. You have evolved as a research scientist, and now you can grow as an educator. Both have setbacks and growing pains.

This perspective will allow you to view yourself less severely as you undertake, or consider undertaking, new activities. Activities in new areas where failure seems likely are often approached tentatively by even the most competent professionals. You may dread failing your own high expectations or looking foolish to yourself or others. This puts considerable pressure on you to do everything right the first time. However, one of the fastest ways to learn quickly is to miss the mark, much as you would do when learning a new language. Falling short should be viewed not as evidence that you are lacking the right stuff to perform in a new environment, but as a vehicle to help you understand which new skills and approaches are needed. Contrary to the NASA maxim, failure should be an option for you, as it is necessary for your growth and the development and refinement of new skills. Keep trying in new ways and with some self-awareness! Things will not always work well in the beginning. Reflect on your efforts, and be open to improvements, realizing that you are adding value, even if you are not meeting your own high expectations. Be authentic, do your best, and seek feedback that can help you in any course corrections.

This perspective is also useful in encouraging girls and students in other groups who do not strongly identify with science and who feel they may fail in science or not have the “right stuff” for science [9]. Encouraging a positive perspective toward experimentation and imperfect results can help students who are uncomfortable with science feel better about taking the first steps. Many students feel that science is too hard for them or that they are not suited for learning and doing science [10]. Please don’t reinforce these concerns by trying to impress them with how much you know or how smart they need to be to understand science. Your perspective should be one of encouragement.

A more tolerant outlook can help scientists be more encouraging to students. Science should not be treated as the province for the most intelligent or most accomplished but rather as an intriguing subject area where interest and skills can be developed. Concepts that are difficult today can be mastered with some effort. This process will lead to them developing more confidence when confronted with learning in a new area.

In summary, adopting a perspective that allows for improvement and growth can make you feel more comfortable when you engage in new educational activities. It can also help you convey to students that they can foster and cultivate their own interest in science even if they feel that they are quite different from you or are not as smart as you. Many students feel impossibly distanced from the possibility of understanding science. When a student says “I’m not a science person,” encourage them and communicate the excitement that you feel for your field. Your job is not to make them into scientists but to encourage them as curious science learners.

## Conclusions

These 10 rules can help you as a scientist be happier and more productive in your educational contributions. These principles encourage a more collaborative and cooperative approach by scientists to serving the science education system and its needs. We have stressed the need for developing a high degree of intentionality to encourage the best and most efficient use of your precious time. The approach we advocate is the perspective of the Roman Stoic philosopher Seneca: “If one does not know to which port one is sailing, no wind is favorable.”

The opposite approach is described by Lewis Carroll in Alice’s question to the Cheshire Cat:

Alice: Would you tell me, please, which way I ought to go from here?

The Cheshire Cat: That depends a good deal on where you want to get to.

Alice: I don’t much care where.

The Cheshire Cat: Then it doesn’t much matter which way you go.

We are confident that you, as a professional scientist, can be successful in partnering with science educators. Your deeper understanding and appreciation of the many challenges that informal and formal educators face will serve you well as you bring your commitment, ingenuity, and scientific expertise to your efforts.
